# Plantagoside Alleviate Hepatic Inflammation, Oxidative Stress and Histopathological Damage in D‐Galactose‐Induced Senescent Mice by Modulating Purine Metabolic Pathways

**DOI:** 10.1002/fsn3.71153

**Published:** 2025-10-31

**Authors:** Jinjia Liu, Yuning Zhang, Furong Lv, Chengming Wang, Jixiang Wang, Lijuan Li, Jinqiang Wu, Lina Lai

**Affiliations:** ^1^ Department of Biochemistry, Basic Medical College Changzhi Medical College Changzhi Shanxi China; ^2^ School of Pharmacy Changzhi Medical College Changzhi Shanxi China; ^3^ Shanxi Provincial Department‐Municipal Key Laboratory Cultivation Base for Quality Enhancement and Utilization of Shangdang Chinese Medicinal Materials Changzhi China; ^4^ Clinical Medicine The Second Hospital of Shanxi Medical University Taiyuan Shanxi China; ^5^ College of Life Sciences and Medicine Zhejiang Sci‐Tech University Zhejiang Hangzhou China; ^6^ Department of Epidemiology and Health Statistics of Public Health and Preventive Medicine Changzhi Medical College Changzhi China; ^7^ School of Nursing Changzhi Medical College Changzhi Shanxi China; ^8^ Department of Histology and Embryology, Basic Medical College Changzhi Medical College Changzhi Shanxi China

**Keywords:** D‐galactose‐induced senescence, hepatoprotection, oxidative stress and inflammatory response, plantagoside, purine metabolism

## Abstract

In this study, we aimed to investigate the hepatoprotective effects of plantagoside (PLA) against D‐galactose (D‐gal)‐induced liver injury in aged mice. PLA treatment significantly alleviated the D‐gal‐induced body weight loss and the elevated liver index in mice, and effectively reduced oxidative stress, inflammatory response, and liver tissue damage in vivo. Transcriptomic analysis identified 513 differentially expressed genes (DEGs) regulated by PLA, and enrichment analysis revealed that these genes were primarily associated with the PI3K‐Akt signaling pathway and purine metabolism. Metabolomic analysis revealed that PLA regulates purine metabolism by modulating key metabolites, such as hypoxanthine and cAMP. Integrated omics analysis suggested that PLA exerts protective effects by regulating purine metabolism, thereby enhancing antioxidant capacity and suppressing inflammation. In conclusion, PLA significantly alleviated D‐gal‐induced liver injury, reduced oxidative stress, and attenuated inflammation by regulating purine metabolism, thereby demonstrating its potential as an anti‐aging and hepatoprotective drug and providing a new theoretical basis for the treatment of age‐associated liver diseases.

## Graphical abstract

Protective role of plantagoside in D‐galactose‐induced hepatic aging through regulation of purine metabolism.

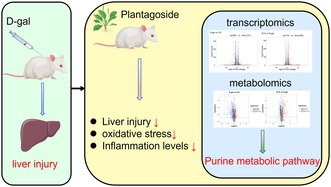

## 1 Introduction

In aging‐associated liver injury, oxidative stress directly damages hepatocytes and promotes inflammation by inducing DNA damage, activating cellular senescence pathways, disturbing antioxidant homeostasis, and exacerbating lipid metabolism disorders. Chronic inflammation further exacerbates liver injury by activating immune cells, releasing pro‐inflammatory factors, inducing hepatocyte necrosis, and promoting fibrosis. The two interact to form a vicious circle, and together promote the progression of aging‐associated liver injury (Aljobaily et al. 2020; Pedroza‐Diaz et al. 2022). The liver plays a key role in the aging process of the whole body, and a decline in its metabolic and immune functions not only affects its own health but also triggers systemic metabolic disorders and chronic inflammation (Yao et al. 2023). Therefore, intervention strategies targeting hepatic aging are important to delay systemic aging and prevent chronic diseases. The D‐galactose (D‐gal)‐induced senescence model has significant advantages in mimicking natural aging, and its high efficiency, rapidity, and low cost make it an important tool for studying aging mechanisms and screening anti‐aging interventions; its well‐defined induction mechanism and wide applicability provide a reliable experimental platform for aging research (Wang et al. 2022). The D‐gal‐induced liver aging model is not only used to study the basic mechanisms of liver aging but also to explore the pathogenesis of aging‐related liver diseases, and provides an important experimental basis for the development of anti‐aging drugs and interventions (Azman et al. 2021).

Altered purine metabolism is a hallmark of aging, and its imbalance leads to impaired cellular energy metabolism, cell cycle arrest, heightened inflammatory responses, and cellular dysfunction, thereby driving the onset and progression of aging (Tabata et al. 2023). During liver aging, purine metabolism plays an important role in regulating oxidative stress, energy metabolism, and inflammatory responses; its disruption can exacerbate oxidative damage and cellular dysfunction, whereas optimizing purine metabolism can reduce oxidative stress and slow down liver aging (Lu et al. 2023). Plantain ( *Plantago asiatica* L.) is an herb native to East Asia that has been used in traditional Asian medicine for more than 2000 years to treat liver disorders, stomach disorders, and inflammation of the urinary system (Wen et al. 2022). Plantain extract has significant anti‐inflammatory, antioxidant, and cell migration‐promoting abilities; it reduces the level of inflammatory factors by inhibiting the TLR4‐MyD88‐NF‐kB signaling pathway and participates in purine metabolism to reduce the generation and accumulation of uric acid, which effectively relieves renal cell inflammation and protects renal function (Liu et al. 2025). Plantain seed extract has been reported to significantly ameliorate isoproterenol‐induced cardiac hypertrophy in mice, attenuate the impairment of cardiac function, and downregulate the expression of hypertrophic markers by inhibiting excessive autophagy and apoptosis in cardiomyocytes, demonstrating a protective effect against cardiac hypertrophy (Fan et al. 2021). Previous studies had reported significant antioxidant and anti‐inflammatory activities of plantain seed extract plantagoside (PLA), which inhibits UV‐induced MMP‐1 expression, reduces skin wrinkle formation, and maintains skin collagen content, thus showing potential for preventing skin photoaging (Kim et al. 2015). PLA is a flavanone glucoside isolated from plant seeds. Although it has been shown to be an important active ingredient in plantains, with significant antioxidant, anti‐inflammatory, and antitumor activities, research on it is still in the preliminary stage, and further experiments would be required to verify its effectiveness (Wen et al. 2022).

With consistent aging of the population, exploring anti‐aging strategies and mechanisms has become a hot research topic, and the anti‐aging effects and protective mechanisms of PLA in the liver remain to be explored. Here, we combined transcriptomic and metabolomic techniques to investigate the protective effects and molecular mechanisms of PLA on the liver of a D‐gal‐induced senescence model, aiming to provide a new theoretical basis and potential targets for the development of anti‐aging drugs.

## 2 Materials and Methods

### 2.1 Materials and Reagents

Eight‐week‐old specific‐pathogen‐free (SPF) male BALB/c mice (Bo‐Htay et al. 2018), with body weight 20.324 ± 1.58 g, were purchased from SiPeiFu Biotechnology Co. (Beijing, China). The mice were housed in a well‐ventilated, clean animal house with a 12‐h light–dark cycle, an ambient temperature of 20°C–25°C, and a relative humidity of 50%–60%. The mice were acclimatized to the environment for 1 week prior to the experiment. The experimental protocol was approved by the Animal Protection and Utilization Committee of the Changzhi Medical College, Shanxi, China. The animals were maintained in strict compliance with the ethical guidelines established by the World Organization for Animal Health. Standard feed (Co60 radiation‐sterilized) was purchased from Open Source Animal Feed (Changzhou) Co. PLA (purity ≥ 98.0%) was purchased from Shanghai Aladdin Biochemical Science and Technology Co. D‐gal was purchased from Sigma‐Aldrich (St. Louis, MO, USA), and PEG 300 and hematoxylin and eosin (H&E) were purchased from Beijing Solarbio Biotechnology Co. Ltd. (Beijing, China). Superoxide dismutase (SOD), glutathione (GSH), malondialdehyde (MDA), tumor necrosis factor α (TNF‐α), interleukin 6 (IL‐6), interleukin 1β (IL‐1β), alanine aminotransferase (AST), and alanine aminotransferase (ALT) were purchased from Xiamen Lunchangshuo Bio‐technology Co. Antibody of rabbit anti β‐actin (l102) was purchased from Bioworld Technology Inc. (Nanjing, China). Antibodies of rabbit anti PPAT (A6698), rabbit anti Paics (A6450), rabbit anti Adcy (A9850), rabbit anti P21 (A19094) and rabbit anti P53 (A19585) were purchased from ABclonal Technology Co. Ltd. (Wuhan, China). HRP Anti‐Rabbit IgG (BA1054) was purchased from Wuhan Boster Biological Technology Co. Ltd. (Wuhan, China).

### 2.2 Animal Experiment Design

Fifty mice were randomly divided into five groups of 10 mice each, namely the blank control group (Ctrl), the aging model group (D‐gal: 120 mg/kg (Qi et al. 2020)), low concentration PLA‐treated group (PLA‐L: 120 mg/kg D‐gal + 50 mg/kg PLA), high concentration PLA‐treated group (PLA‐H: 120 mg/kg D‐gal + 200 mg/kg PLA), and vitamin C (VC) positive control treatment group (VC: 120 mg/kg D‐gal +100 mg/kg VC (Shen et al. 2022)). The PLA concentration was selected based on the results of a preliminary experiment. Referring to the reagent instructions, PLA was dissolved in an aqueous solution containing 40% PEG 300 (dispersant) and administered by gavage according to the body weight of the mice. D‐gal was dissolved in 0.9% saline and administered by subcutaneous injection to the back of the neck of mice once daily for 8 weeks. Mice in the control group were injected with an equal amount of 0.9% saline and gavaged with an equal amount of dispersant. Mice in the D‐gal group were gavaged with an equal amount of the dispersant. The injections were completed at 10:00 a.m., and the gavage was completed at 6:00 p.m. each day. After 24 h of the last dose, and following a 12‐h fast, the mice were anesthetized with ether and euthanized. The liver and serum of the mice were rapidly separated, and the weight of the livers (mg) was measured and compared with the body weights of the mice (g) to calculate the liver index. A portion of the liver tissue was preserved in 4% formaldehyde while the rest was frozen in a refrigerator at −80°C for subsequent experiments.

### 2.3 Biochemical Analysis

At first, mice were sacrificed, and blood samples were collected from the retro‐orbital venous plexus. Subsequently, the collected blood samples were centrifuged at 3500 × g for 10 min at 4°C to separate the serum. The relevant serum indices were determined separately using assay kits. In addition, mouse liver tissues were homogenized. Liver tissues were prepared as a 10% (w/v) homogenate in saline under ice‐bath conditions and then centrifuged at 2500 × g for 10 min at 4°C to separate the supernatant. The relevant indices of the liver homogenates were determined separately using the corresponding biochemical kits. All steps were performed in strict accordance with the manufacturer's instructions.

### 2.4 H&E Staining

Liver tissue samples were fixed in 4% neutral buffered formaldehyde for 1 day to maintain the structural integrity of the tissue. Subsequently, the tissues were dehydrated and embedded in paraffin and cut into 5‐μm‐thick sections. Next, the sections were deparaffinized with xylene to remove paraffin and restore transparency, and then sequentially hydrated in an aqueous environment using a gradient of ethanol. Thereafter, the sections were stained with hematoxylin for 5–10 min to stain the nuclei blue‐purple; after differentiation and reblue treatment, they were stained with eosin for 1–2 min to make the cytoplasm pink. Finally, the sections were rinsed twice with 70% ethanol to remove all excess dye and then sealed to observe the morphology of the histiocytes under an inverted microscope (Olympus Corporation, Tokyo, Japan) at 20‐ and 40‐fold magnification.

### 2.5 Transcriptomic Analysis of Mouse Liver Tissue

Liver tissue samples from three randomly selected mice in each experimental group were used for transcriptomic analysis. The PLA‐H group was selected for subsequent histological analysis based on the results of previous experiments. Total RNA, extracted from fresh mouse liver tissue, was isolated and purified using TRIzol reagent (Invitrogen) following the manufacturer's instructions. Subsequently, the concentration and purity of RNA were quantified using a NanoDrop ND‐1000 to ensure that the concentration of RNA was greater than 50 ng/μl and the OD_260/280_ ratio was greater than 1.8. The integrity of the RNA was assessed using a Bioanalyzer 2100 (Agilent) to ensure that the RIN value was greater than 7.0. RNA integrity was further verified by denaturing agarose gel electrophoresis. After the above conditions were met, two rounds of purification were performed from 1 μg of total RNA using Dynabeads Oligo (dT) magnetic beads (Thermo Fisher) to specifically capture the mRNAs with poly (A) tails. The captured mRNAs were fragmented using the Magnesium Ion RNA Fragmentation Kit (NEB) at 94°C for 5–7 min. The fragmented RNA was subsequently reverse transcribed to synthesize cDNA using SuperScript II Reverse Transcriptase (Invitrogen). cDNA was synthesized using *Escherichia coli* DNA Polymerase I (NEB), RNase H (NEB), and dUTP solution (Thermo Fisher Scientific) to obtain the second‐stranded DNA with U labeling. An A‐base was added to the end of the DNA duplex for ligation to the junction with a T‐base. Fragment size selection and purification were performed by AMPureXP magnetic beads, resulting in the construction of a cDNA library with an average insert fragment size of 300 ± 50 bp. Finally, 2 × 150 bp double‐end sequencing (PE150) was performed using an Illumina Novaseq 6000 sequencing platform.

Data analysis was performed using the fastp software for quality control of the raw FASTQ format data downstream, removing sequences containing splice contamination, low‐quality bases, and indeterminate bases, and verifying the quality of the sequencing data. Subsequently, the sequencing data were aligned to the mouse reference genome ( *Mus musculus* , e.g., GRCm39 version) using HISAT2 software to generate the alignment file in the bam format. Next, the genes or transcripts were assembled using StringTie software and quantitatively analyzed using the fragments per kilobase per million (FPKM) method, calculated as follows: FPKM = [total_exon_fragments/mapped_reads (millions) × exon_length (kB)]. Transcripts from all samples were merged using gffcompare software to reconstruct a comprehensive transcriptome. The expression levels of all transcripts in the final transcriptome were estimated using StringTie. Genes differentially expressed between samples were analyzed using the edgeR package. mRNAs with a multiplicity of difference greater than 2 or less than 0.5 and a *p* value less than 0.05 were selected as differentially expressed genes (DEGs) and were analyzed by Gene Ontology (GO) and Kyoto Encyclopedia of Genes and Genomes (KEGG) enrichment using DAVID software to further understand their functions in the biological processes and signaling pathways involved.

### 2.6 Metabolomic Analysis of Mouse Liver Tissue

Liver tissue samples from three randomly selected mice in each experimental group were used for metabolomic analysis. After thawing the collected mouse liver tissue samples on ice, 20 μL from each sample was taken and 120 μL of pre‐cooled 50% methanol buffer was added for metabolite extraction. The mixture was then vortexed for 1 min, incubated at room temperature for 10 min, and stored at −20°C overnight. After centrifugation (4000 × g, 20 min), the supernatant was transferred to a 96‐well plate and stored at −80°C for subsequent liquid chromatography‐mass spectrometry (LC–MS) analysis. Meanwhile, mixed quality control (QC) samples were prepared by mixing 10 μL of each extraction mixture.

A SCIEX TripleTOF 5600 Plus high‐resolution tandem mass spectrometer combined with an ultra‐performance liquid chromatography (UPLC) system was used for LC–MS analysis. An ACQUITY UPLC T3 column (100 mm × 2.1 mm, 1.8 μm, Waters, UK) was used for reversed‐phase separation. The mobile phase consisted of solvent A (water containing 0.1% formic acid) and solvent B (acetonitrile containing 0.1% formic acid), and the gradient elution conditions were as follows: 5% solvent B for 0–0.5 min, 5%–100% solvent B for 0.5–7 min, 100% solvent B for 7–8 min, 100%–5% solvent B for 8–8.1 min, and 5% solvent B for 8.1–10 min, with a flow rate of 0.4 mL/min, and the column temperature maintained at 35°C. The ion spray float voltage was set to 5 kV in positive ion mode and −4.5 kV in negative ion mode. Mass spectrometry data were collected in IDA mode with a TOF mass range of 60–1200 Da. Mass calibration was performed every 20 samples, and QC samples were analyzed every 10 samples to assess the stability of the LC–MS.

The collected LC–MS data were preprocessed using the XCMS software. The raw data files were converted to mzXML format and processed using the XCMS, CAMERA, and metaX toolboxes (in R software). Each ion was identified based on the combined retention time and m/z information, and the intensity of each peak was recorded to generate a three‐dimensional matrix containing arbitrarily assigned peak indices (retention time–m/z pairs), sample names (observations), and ion intensity information (variables). The information was then matched against internal and public databases, using the KEGG and HMDB open‐access databases, to annotate the metabolites by matching the exact molecular mass data (m/z) to those in the databases within a threshold of 10 ppm. Peak intensity data were further preprocessed using metaX to remove features detected in less than 50% of QC samples or 80% of test samples, and the values of missing peaks were extrapolated using the k‐nearest‐neighbor algorithm to improve the data quality. PCA was performed using a preprocessed dataset to detect outliers and batch effects. A robust QC‐based LOESS signal correction was fitted to the QC data to minimize the drift of signal intensity over time based on the injection order. In addition, the relative standard deviations of metabolite signatures were calculated for all QC samples, removing signatures with standard deviations greater than 30%. The group dataset was normalized prior to analysis using the probability quotient normalization algorithm for all samples, and the QC samples were used for QC‐robust spline correction. *p* values were analyzed using the Student's t‐test and corrected for multiple testing using the FDR (Benjamini‐Hochberg) method for the selection of different metabolites. In addition, supervised PLS‐DA analyses were performed using metaX to identify the more specific differences between groups, and a VIP cutoff value of 1.0 was set to select important features.

### 2.7 Co‐Analysis of Transcriptional Combinatorial Metabolomics in Mouse Liver Tissue

Pathways with KEGG‐enriched pathway enrichment coefficients (*p* value < 0.05) for DEGs and metabolites were selected for Wayne's analysis to obtain the shared enriched pathways. The pathways with the highest sum of enriched DEGs and differential metabolites were mapped to the corresponding KEGG pathway maps, and the nodes with significant downregulation or upregulation of gene or metabolite expression were labeled in blue and red, respectively, to understand the relationship between genes and metabolites better.

### 2.8 Western Blotting

Liver tissues were homogenized in ice‐cold RIPA lysis buffer supplemented with protease and phosphatase inhibitors and incubated on ice for 20 min. The lysates were centrifuged at 12,000 g for 15 min at 4°C to obtain the supernatant containing total protein. Protein concentrations were quantified using the BCA assay. Equal amounts of protein were denatured in loading buffer at 95°C for 5 min, separated by SDS‐PAGE, and transferred onto PVDF membranes. Prior to transfer, the PVDF membranes were activated with methanol. After transfer, the membranes were blocked with 5% skim milk in TBST for 2 h at room temperature. Primary antibodies were applied and incubated overnight at 4°C. Following three washes with TBST (10 min each), membranes were incubated with HRP‐conjugated secondary antibodies for 2 h at room temperature. Protein bands were visualized using an enhanced chemiluminescence (ECL) detection reagent and imaged with a Bio‐Rad ChemDoc system. Densitometric analysis was performed using ImageJ software.

### 2.9 Statistical Analysis

All the data are presented as means ± SEM. Statistical analyses were performed using SPSS (version 22.0; IBM Corp., Armonk, NY, USA). Differences between the Ctrl and D‐gal groups were analyzed using the Student's *t*‐test. For multiple group comparisons, one‐way ANOVA was performed, followed by Dunnett's post hoc test to determine inter‐group differences of PLA‐treated groups relative to the D‐gal group. A *p* < 0.05 was considered statistically significant.

## 3 Results

### 3.1 Effect of PLA Addition on the Body Weight and Liver Index of D‐Gal‐Induced Mice

**FIGURE 1:**
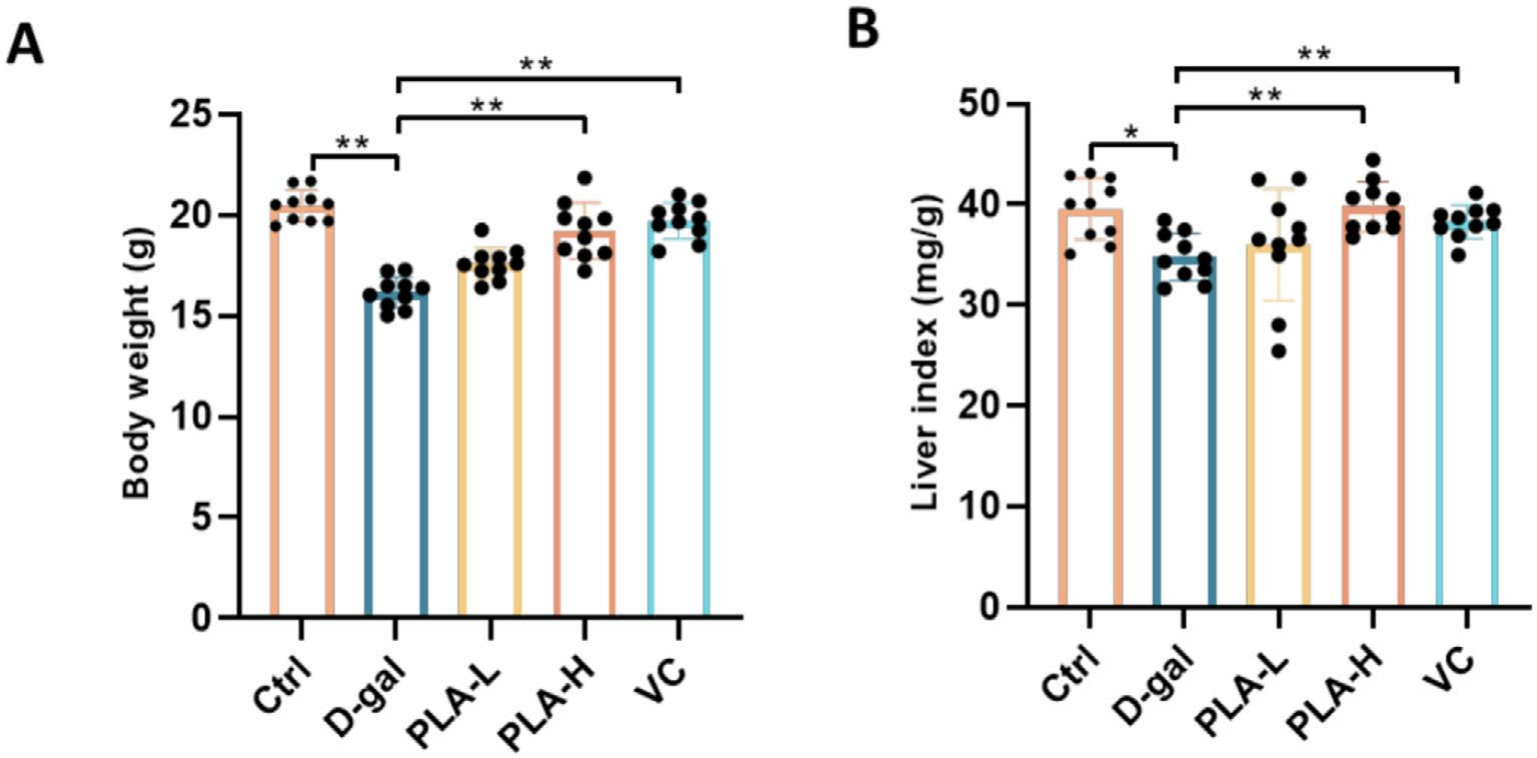
Effect of PLA addition on body weight and liver index in D‐gal‐induced mice. (A) Body weight. (B) Liver index.

Treatment of senescent mice with different concentrations of PLA by gavage resulted in a significant difference in their body weights in the different treatment groups (Figure 1A). The body weight of the D‐gal group was significantly lower than that of the Ctrl group (*p* < 0.01), indicating that D‐gal treatment reduced the body weight of mice. After PLA‐L and PLA‐H treatments, the body mass showed a certain degree of recovery, indicating that PLA treatment had a mitigating effect on D‐gal‐induced body mass reduction and that the effect of high‐dose PLA‐H was more significant (*p* < 0.01). Among the different groups of mice, the liver indices of the D‐gal group were significantly higher than those of the Ctrl group (*p* < 0.01), suggesting that D‐gal treatment resulted in a significant increase in liver indices (Figure 1B). The latter in the PLA‐L and PLA‐H treatments showed a reversal of the decreasing trend, indicating that PLA treatment was capable of partially reversing the elevation of the liver indices induced by D‐gal, and the recovery effect of the PLA‐H group was closer to that of the Ctrl group (*p* < 0.01). The results suggested that PLA could alleviate both D‐gal‐induced body mass loss and liver index elevation and exhibit potential hepatoprotective and anti‐injury effects.

### 3.2 Effect of PLA Addition on D‐Gal‐Induced Oxidative Stress in Mouse Liver

**FIGURE 2:**
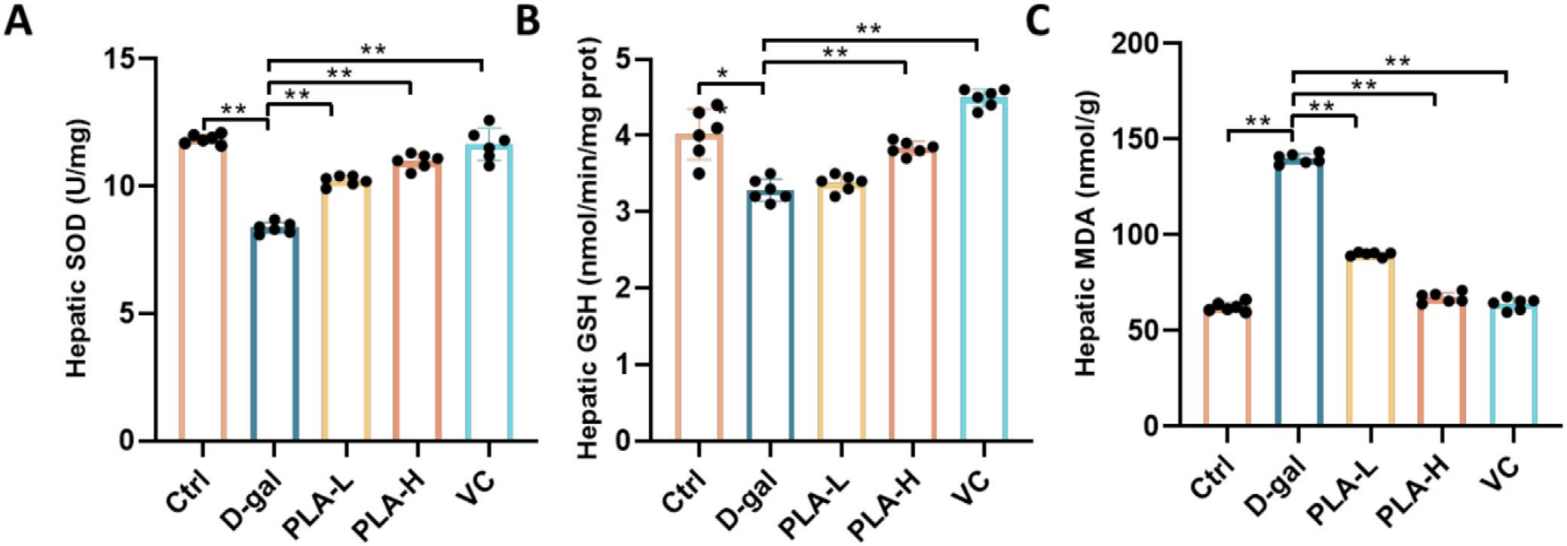
Effect of PLA addition on oxidative stress in the liver of D‐gal‐induced mice. (A) SOD levels. (B) GSH level. (C) MDA level.

The liver SOD activities in the mice in different treatment groups were found to be significantly lower in the D‐gal group than in the Ctrl group (*p* < 0.01), indicating that the D‐gal treatment reduced the antioxidant capacity of the liver in mice (Figure 2A). After treatment with PLA‐L and PLA‐H, SOD activity showed some degree of recovery, indicating that PLA treatment mitigated the D‐gal‐induced decrease in SOD activity and that the effect of high‐dose PLA‐H was more significant (*p* < 0.01) (Figure 2A). The hepatic reduced glutathione (GSH) content of mice in the different treatment groups also showed significant differences (Figure 2B). It was significantly lower in the D‐gal group than in the Ctrl group (*p* < 0.01), suggesting that D‐gal treatment weakened the antioxidant defense capacity of the mouse liver. After PLA‐L and PLA‐H treatments, the GSH content showed a reversal of the trend, indicating that PLA treatment partially reversed the D‐gal‐induced decrease in GSH content, and the recovery effect of the PLA‐H group was closer to that of the control group (*p* < 0.01). Additionally, a significant difference in hepatic malondialdehyde (MDA) content was observed across the different treatment groups (Figure 2C). The MDA content in the D‐gal group was significantly higher than that in the Ctrl group (*p* < 0.01), indicating that D‐gal treatment significantly increased the hepatic lipid peroxidation levels. After PLA‐L and PLA‐H treatments, the MDA content showed a reverse downward trend, indicating that PLA treatment partially reversed the D‐gal‐induced elevation in MDA content, and the recovery effect of the PLA‐H group was closer to that of the Ctrl group (*p* < 0.01). The results collectively indicated that PLA has both antioxidant and protective effects.

### 3.3 Effect of PLA Addition on Serum and Liver Inflammatory Cytokines and Hepatic Injury Markers in D‐Gal‐Induced Mice

**FIGURE 3:**
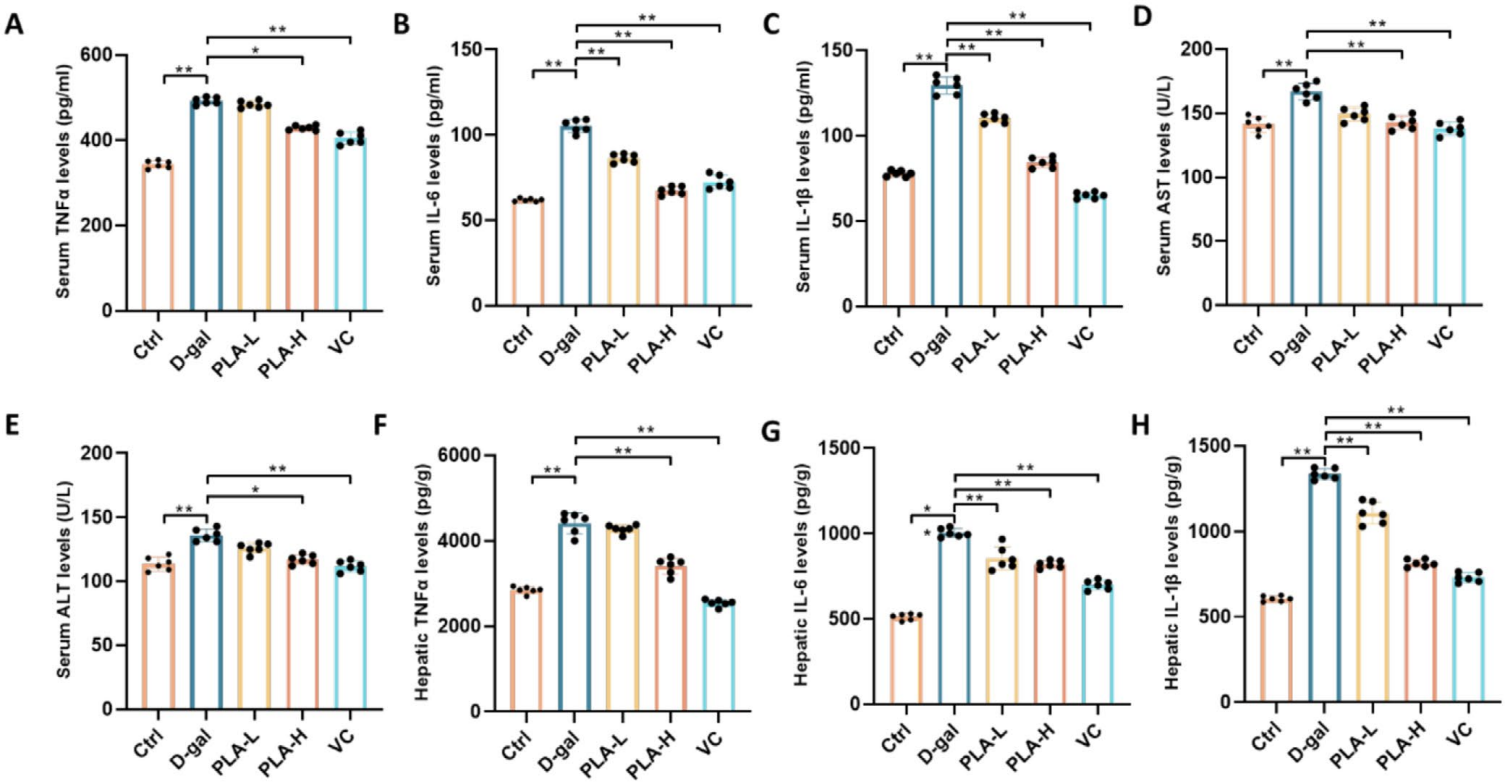
Effect of PLA addition on serum and liver inflammatory cytokine levels in D‐gal‐induced mice. (A) Serum TNFα levels. (B) Serum IL‐6 levels. (C) Serum IL‐1β levels. (D) Serum AST levels. (E) Serum ALT levels. (F) Hepatic homogenate TNFα levels. (G) Hepatic homogenate IL‐6 levels. (H) Hepatic homogenate IL‐1β levels.

The serum TNF‐α level (Figure 3A) and liver TNF‐α level (Figure 3F) of mice in the D‐gal group were significantly higher than those in the Ctrl group (*p* < 0.01), suggesting that D‐gal treatment induced systemic and hepatic inflammatory responses. After treatment with PLA‐L and PLA‐H, both serum and liver TNF‐α levels showed a trend of reversed decrease, indicating that PLA treatment partially reversed the D‐gal‐induced TNF‐α elevation, and the recovery effect was more significant at the high dose of PLA‐H (*p* < 0.01). The serum IL‐1β levels (Figure 3B) and liver IL‐1β levels (Figure 3H) of mice in the D‐gal group were both significantly higher than those in the Ctrl group (*p* < 0.01). After treatment with PLA‐L and PLA‐H, the serum and liver IL‐1β levels showed a trend of reversed decrease, indicating that PLA treatment could partially reverse the D‐gal‐induced IL‐1β elevation, and the recovery effect of the PLA‐H group was closer to that of the Ctrl group (*p* < 0.01). The serum IL‐6 levels (Figure 3C) and liver IL‐6 levels (Figure 3G) of mice in the D‐gal group were significantly higher than those in the control group (*p* < 0.01). After PLA‐L and PLA‐H treatments, serum and liver IL‐6 levels showed a trend of reversal of the decrease, indicating that PLA treatment partially reversed D‐gal‐induced IL‐6 elevation, and the recovery effect of the PLA‐H group was closer to that of the Ctrl group (*p* < 0.01). Serum AST (Figure 3D) and ALT (Figure 3E) levels were significantly higher in the D‐gal group than in the Ctrl group (*p* < 0.01). After treatment with PLA‐L and PLA‐H, the serum AST and ALT levels showed a trend of reverse decrease, indicating that PLA treatment could partially reverse D‐gal‐induced AST and ALT elevation, and the recovery effect of the PLA‐H group was closer to that of the Ctrl group (*p* < 0.01). These results indicate that PLA‐L and PLA‐H treatments reversed D‐gal‐induced liver damage to a certain extent, showing that PLA has anti‐inflammatory and protective effects.

### 3.4 Effect of PLA Addition on the Histopathology of Liver in D‐Gal Induced Mice

**FIGURE 4:**
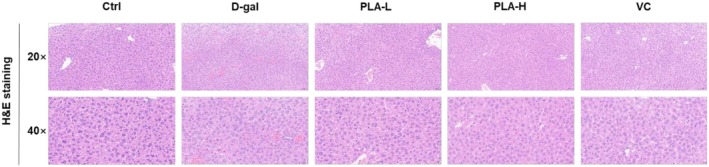
Effect of PLA on the histopathology of liver in D‐gal‐induced mice.

The effects of PLA on the histopathology of D‐gal‐induced mouse livers were observed from the histopathological sections of the livers (Figure 4); the livers of mice in the Ctrl group were intact, with hepatic lobules arranged in an orderly manner and no obvious pathological alterations, whereas those in the D‐gal‐treated group showed significant hepatic injury features, including dilated hepatic sinusoids, disordered arrangement of hepatocytes, mild edema of hepatocytes, hepatocytes occasionally showing intranuclear inclusion bodies, and more vascular sludge. Compared to the D‐gal group, the PLA‐L group significantly reduced the degree of hepatocellular injury, reduced intranuclear inclusion bodies and vascular stasis, and promoted partial repair of the tissue structure. The PLA‐H group showed superior hepatoprotective effects, with the structure of the hepatic lobules basically restored to normal, and the histological characteristics close to those of the normal control group. The results confirmed that PLA intervention could reverse D‐gal‐induced hepatic tissue injury in a dose‐dependent manner and that the effect of high‐dose PLA in restoring the structural and functional integrity of the liver was particularly significant.

### 3.5 Effect of PLA Addition on the Liver Transcriptome of D‐Gal‐Induced Mice

**FIGURE 5:**
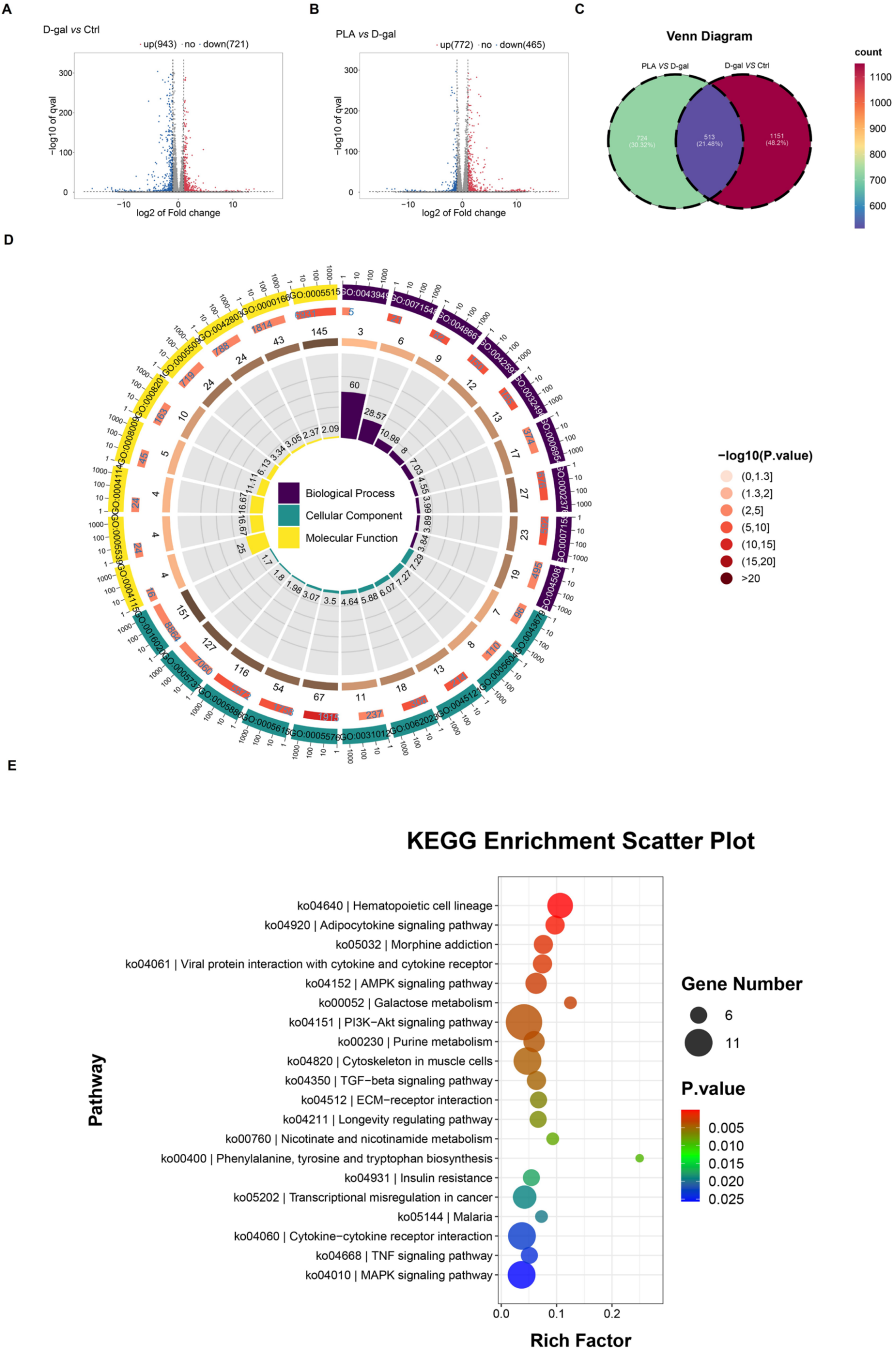
Effect of added PLA on the transcriptome of D‐gal‐induced mouse liver. (A) Volcano plot of differentially expressed genes in the D‐gal group versus the Ctrl group. (B) Volcano plot of differentially expressed genes in PLA group vs. D‐gal group. (C) Wayne plots of the shared differentially expressed genes of the Ctrl group, D‐gal group and PLA group. (D) GO analysis of shared differentially expressed genes in Ctrl group, D‐gal group and PLA group. (E) KEGG analysis of shared differentially expressed genes in Ctrl group, D‐gal group and PLA group.

The mechanism of the hepatoprotective effect of PLA on D‐gal‐treated mice was explored using transcriptomics, and the experimental results revealed a total of 1664 DEGs in the livers of mice in the D‐gal group than in the Ctrl group; of them, 943 were upregulated and 721 were downregulated (Figure 5A). A total of 1237 DEGs were detected in the livers of mice in the PLA group than in the D‐gal group, including 772 upregulated and 465 downregulated genes (Figure 5B). By performing Venn analysis of these DEGs (Figure 5C), 513 genes were identified to be common among the three groups, including target genes for PLA to exert hepatoprotective effects. Therefore, we performed GO (Figure 5D) and KEGG (Figure 5E) enrichment analyses of these shared genes to further clarify their functions. GO enrichment analyses indicated that the functions of these genes might be related to the regulation of cAMP‐mediated signaling (GO:0043949), 3′,5′‐cyclic‐AMP phosphodiesterase activity (GO:0004115), and axon terminus (GO:0043679). KEGG enrichment analysis indicated that PLA could exert hepatoprotective effects through the hematopoietic cell lineage, adipocytokine signaling pathway, AMPK signaling pathway, Purine metabolism, and PI3K‐Akt signaling pathway.

### 3.6 Effect of PLA Addition on the Hepatic Metabolome of D‐Gal‐Induced Mice

**FIGURE 6:**
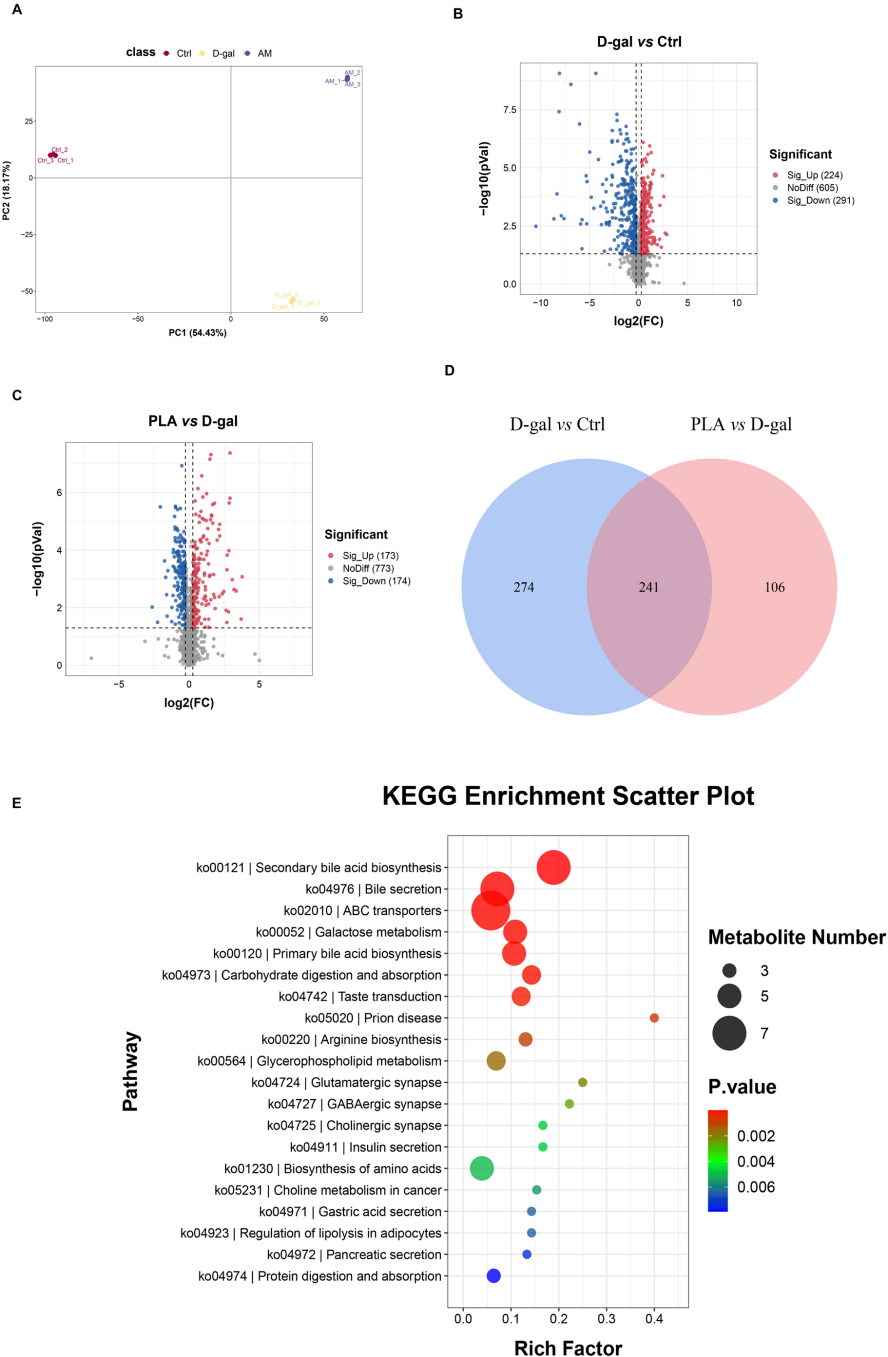
Effects of added PLA on the liver metabolome of D‐gal‐induced mice. (A) PCA analysis of Ctrl group, D‐gal group, and PLA group. (B) Differential metabolite volcano plots of D‐gal group vs. Ctrl group. (C) Differential metabolite volcano plots of PLA group vs. D‐gal group. (D) Wayne analysis of shared differential metabolites of Ctrl group, D‐gal group, and PLA group. (E) KEGG analysis of shared differential metabolites of Ctrl group, D‐gal group & PLA group.

The mechanism underlying the hepatoprotective effect of PLA in D‐gal‐treated mice was further explored by metabolomics based on transcriptome analysis. PCA revealed significant differences in the liver metabolic profiles of mice in the Ctrl, D‐gal, and PLA groups (Figure 6A). By comparing the different groups, a total of 515 differential metabolites were detected in the D‐gal group than in the Ctrl group, of which 224 were upregulated in expression and 291 were downregulated (Figure 6B). Compared to those in the D‐gal group, 347 differential metabolites were detected in the PLA group, of which 173 were upregulated and 174 were downregulated (Figure 6C). To identify the differential metabolites that could be regulated by PLA, we performed Venn analysis of the differential metabolites mentioned above (Figure 6D), and obtained 241 differential metabolites in the three groups. KEGG analysis of these 214 differential metabolites revealed that they could be involved in secondary bile acid biosynthesis, bile secretion, ABC transporters, galactose metabolism, and primary bile acid biosynthesis, among other biological processes (Figure 6E).

### 3.7 Combined Analysis of Transcriptomic and Metabolomic Data of D‐Gal‐Induced Mouse Liver Upon PLA Addition

**FIGURE 7:**
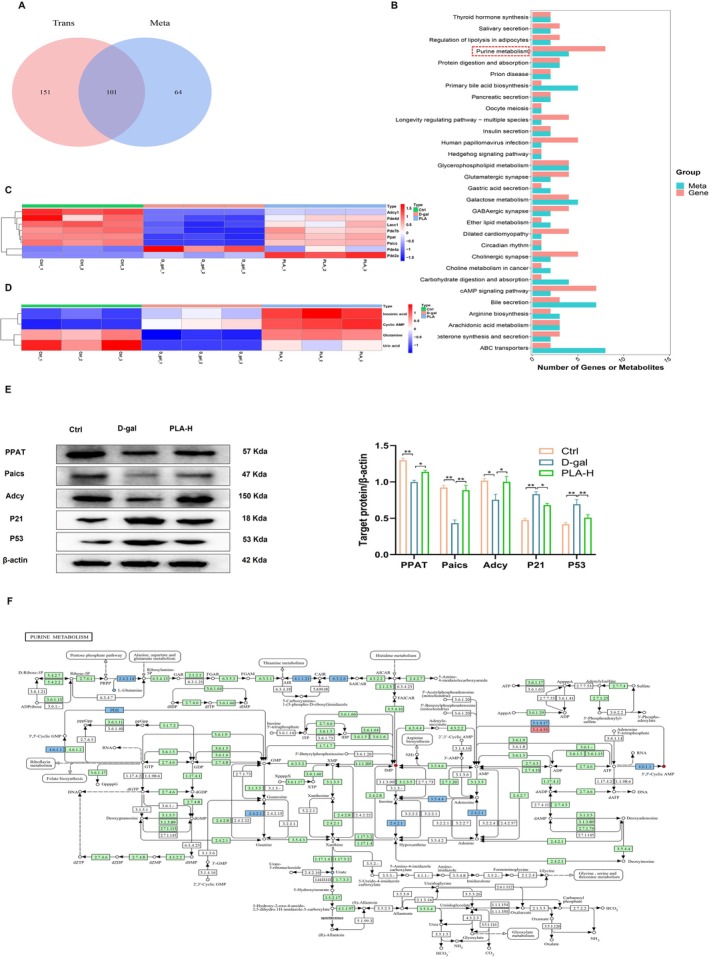
Combined analysis of transcriptomics and metabolomics of D‐gal‐induced mouse liver by addition of PLA. (A) KEGG analysis of differential genes and differential metabolites for shared pathway Wayne plots. (B) Pathway bar graph of KEGG enrichment analysis of differential genes and differential metabolites in the first 30. (C) Heatmap of the expression of differential genes shared by Ctrl group, D‐gal group, and PLA group in purine metabolism pathway. (D) Heatmap of the expression of differential metabolites shared by the Ctrl group, D‐gal group, and PLA group in the purine metabolism pathway. (E) PLA regulates the expression and quantitative analysis of purine metabolism pathway and senescence‐related proteins. (F) KEGG pathway map of purine metabolism. (F) Blue markers (squares represent genes, circles represent metabolites) in the graphs indicate differential genes or differential metabolites whose expression is downregulated in the D‐gal group compared to the Ctrl group and upregulated in the PLA group compared to the D‐gal group; red markers indicate differential genes or differential metabolites whose expression is upregulated in the D‐gal group compared to the Ctrl group and downregulated in the PLA group compared to the D‐gal group.

Transcriptomic and metabolomic data were jointly analyzed to clarify the mechanism underlying the hepatoprotective effects of PLA in D‐gal‐treated mice. Wayne's analysis of KEGG‐enriched pathways for the three groups of shared DEGs and metabolites revealed 101 KEGG pathways (Figure 7A). Among them, the sum of the number of differential genes and metabolites enriched in purine metabolism was the highest (Figure 7B). Therefore, we hypothesized that purine metabolism (mmu00230) might be closely related to the hepatoprotective effects of PLA; eight DEGs were enriched in purine metabolism, and PLA was effective in reversing the changes in the expression of these genes caused by D‐gal (Figure 7C). Four differentially expressed metabolites were enriched in purine metabolism, and PLA was effective in reversing the D‐gal‐induced changes in the levels of these metabolites (Figure 7D). These genes or metabolites were found upstream and downstream of purine metabolism, and play important roles in the regulation of purine metabolism.

We examined the expression of relevant proteins regulating these differential metabolites by Western Blotting and found that, consistent with the results of Figure 7C, PLA was effective in restoring the expression of PPAT, Paics, and Adcy (*p* < 0.05) (Figure 7E). Further detection of senescence‐associated P21 and P53 revealed that PLA effectively reduced the elevated expression of these proteins caused by D‐gal (*p* < 0.05) (Figure 7E). These results further suggest that PLA may exert hepatoprotective effects by regulating the expression of proteins in the purine metabolism pathway.

## 4 Discussion

PLA showed significant protective effects in a mouse model of D‐gal‐induced liver injury. It effectively ameliorated weight loss and elevated liver index (Figure 1), reduced oxidative stress and inflammatory responses (Figures 2 and 3), and mitigated hepatic histopathological injury (Figure 4). Mechanistic studies suggested that it might exert hepatoprotective effects by modulating purine metabolic pathways (Figure 7). Our results suggested that PLA is a potential hepatoprotective agent with antioxidant, anti‐inflammatory, and molecular regulatory activities. In neuronal cells, Plantamajoside, a plantain extract, has been shown to reduce oxidative stress‐induced cellular damage, thereby protecting the neuronal cells from oxidative damage and delaying neuronal aging by reducing ROS production (Živković et al. 2017). Further, it could protect skin cells from oxidative damage and slow down skin aging by inhibiting the activation of MAPK and NF‐κB signaling pathways and reducing UV‐induced MMP‐1 expression (Han et al. 2016). In an ischemia–reperfusion injury model, Plantamajoside reduced oxidative stress‐induced apoptosis, protected cardiovascular cells from injury, and delayed cardiovascular cell aging by regulating the Akt/Nrf2/HO‐1 and NF‐κB signaling pathways (Zeng et al. 2022). Overall, the results suggested that PLA and other similar components exhibit significant anti‐aging effects in a variety of cell types, including neuronal, skin, and cardiovascular cells.

The present study investigated the hepatoprotective effects of PLA during aging and revealed its role in the regulation of purine metabolism, which is an important candidate molecule for the development of anti‐aging and hepatoprotective drugs. As a natural product, PLA may have lower toxicity and fewer side effects, making it safer for long‐term applications. Besides its significant antioxidant and anti‐inflammatory effects, PLA exerts additional hepatoprotective effects through the modulation of purine metabolism, via a multi‐target mechanism of action that goes beyond the single function of traditional drugs as an antioxidant or anti‐inflammatory, hence providing possibilities for the development of novel anti‐aging and hepatoprotective drugs. Purine metabolism and aging are closely related, the latter leading to a decreased efficiency of purine metabolism and accumulation of metabolites (Zieliński et al. 2019). Hepatic transcriptomic and metabolomic analyses revealed that PLA plays a hepatoprotective role by regulating purine metabolism (Figure 7). Disturbed purine metabolism could be an important factor in aging‐associated liver injury, and decreased levels of nicotinamide and hypoxanthine could not only weaken the anti‐inflammatory capacity of the liver but also further exacerbate hepatic injury by affecting the metabolic pathways and mitochondrial function (Zhang et al. 2021). In the present study, we found that PLA effectively increased the levels of hypoxanthine (inosinic acid) in the liver of senescent mice (Figure 7D), suggesting that it could exert hepatoprotective effects by regulating the purine metabolic network. Elevated levels of hypoxanthine, a core intermediate in purine nucleotide metabolism, might improve liver function through the following mechanism: hypoxanthine might act as a precursor substance for ATP synthesis to maintain intracellular energy homeostasis through remediation of the synthetic pathway (Frenguelli 2019), which is particularly important for ameliorating aging‐associated mitochondrial dysfunction. We simultaneously observed an increase in the levels of L‐glutamine (Figure 7D–F), phosphoribosyl pyrophosphate amidotransferase (Patat) (Figure 7C,E,F), and phosphoribosylaminoimidazole carboxylase (Paics) in the liver (Figure 7C,E,F), compared to those in senescent mice. These substances and genes were closely related to the metabolism of alanine, aspartate, glutamate, and histidine (Figure 7F), suggesting that PLA could regulate the dynamic balance between purine metabolism and protein synthesis. We found that PLA effectively increased the levels of three key enzymes involved in the purine de novo synthesis pathway (PPAT, Paics, and Adcy) and significantly reduced the expression of senescence‐associated proteins P21 and P53 (Figure 7E). When the purine de novo synthesis pathway is inhibited, ATP depletion triggers energy stress, leading to AMPK activation and stabilization of P53, which in turn transcriptionally upregulates P21, resulting in S‐phase arrest and the onset of cellular senescence (Obajimi et al. 2009). Therefore, PLA may alleviate cellular senescence by promoting purine de novo synthesis to restore intracellular ATP levels, thereby reducing energy stress and suppressing activation of the AMPK‐P53‐P21 signaling pathway.

Further, we found that PLA effectively increased the level of 3′, 5′‐Cyclic AMP (cAMP) in the livers of senescent mice (Figure 7D,F). cAMP activates AMPK via PKA or EPAC, jointly regulating mitochondrial function, inflammation, metabolism, and antioxidant capacity (Aslam and Ladilov 2022). Together, they form a key anti‐aging signaling axis that delays cellular aging processes. Studies have shown that cAMP effectively reduces apoptosis and pro‐inflammatory cytokine production by activating the PKA signaling pathway and inhibiting key signaling pathways, such as ASK1/JNK/p38 and NF‐κB, while upregulating the expression of antioxidant enzymes, which significantly attenuates the cellular damage caused by oxidative stress and inflammatory responses (Cheng et al. 2024). During liver aging, the level of oxidative stress increases significantly, leading to the accumulation of intracellular ROS, which in turn triggers cellular damage and functional decline (Liguori et al. 2018). This oxidative stress not only destroys intracellular biomolecules but also activates a series of pro‐inflammatory signaling pathways, leading to chronic inflammation (Leyane et al. 2022). In contrast, elevated cAMP levels can reduce inflammation in the liver by activating the PKA signaling pathway, inhibiting the over‐activation of signaling pathways such as ASK1/JNK/p38 and NF‐κB, and reducing the production of pro‐inflammatory cytokines (Lai et al. 2019). Additionally, cAMP upregulates the expression of antioxidant enzymes, such as SOD, catalase (CAT), and glutathione peroxidase (GSH‐Px), enhances cellular antioxidant capacity, effectively scavenges intracellular ROS, and maintains intracellular redox homeostasis to alleviate cellular damage caused by oxidative stress (Cheng et al. 2024). This corroborates the antioxidant effects of PLA observed in this study (Figure 2). PLA could maintain normal liver cell function by elevating cAMP levels, reducing cell damage and death, slowing the process of hepatic aging, and providing new strategies and targets for the prevention and treatment of hepatic aging.

The current study revealed that PLA possibly alleviates D‐gal‐induced liver injury by regulating purine metabolism; however, there are certain limitations in the model and in species variability. In the future, the hepatoprotective mechanism of PLA may be further explored using gene knockdown to validate key targets and cross‐species models, optimize dose design, integrate multi‐omics to analyze the metabolic interaction network, and conduct clinical translational research, thereby contributing to its potential application in the treatment of age‐related liver disease. PLA can be used to alleviate D‐gal‐induced liver injury by modulating the purine metabolism‐oxidative stress‐inflammation (PAM‐inflammatory) mechanism. PLA activates the AMPK/PKA signaling pathway by upregulating the levels of hypoxanthine and cAMP, enhances the hepatic antioxidant capacity (SOD and GSH increase, MDA decreases), inhibits the NF‐κB‐mediated inflammatory cascade (TNF‐α, IL‐1β, and IL‐6 downregulation), and repairs the dynamic balance between purine metabolism and protein synthesis, thereby ameliorating hepatic histopathological injury (Figure 7E). This multitarget mechanism of action makes PLA a promising natural anti‐aging drug.

## 5 Conclusion

The present study revealed the significant protective effects of PLA against D‐gal‐induced liver injury in senescent mice. PLA effectively attenuated oxidative stress and inflammatory responses and ameliorated hepatic histopathological injury by modulating purine metabolic pathways. Its multi‐target mechanisms included enhancing antioxidant capacity, inhibiting the production of pro‐inflammatory factors, and repairing the dynamic balance between purine metabolism and protein synthesis. Our findings suggested that PLA could be a highly promising natural anti‐aging and hepatoprotective drug, and provided an important theoretical basis and experimental support for the development of novel anti‐aging interventions.

## Author Contributions

**Jinjia Liu:** conceptualization, methodology, validation, writing – original draft, writing – review and editing, resources, funding acquisition. **Yuning Zhang:** conceptualization, methodology, data curation, writing – original draft, validation. **Furong Lv:** writing – review and editing, validation. **Chengming Wang:** methodology, software, validation. **Jixiang Wang:** validation, data curation. **Lijuan Li:** data curation, validation. **Jinqiang Wu:** validation, software. **Lina Lai:** supervision, writing – review and editing, project administration, funding acquisition.

## Ethics Statement

Ethical approval for this research was granted by the Experimental Animal Ethics Committee of Changzhi Medical College (Identification Number: DW2024153).

## Conflicts of Interest

The authors declare no conflicts of interest.

## Data Availability

The data that support the findings of this study are available from the corresponding author upon reasonable request.
